# Structural Investigation and Energy Transfer of Eu^3+^/Mn^4+^ Co-Doped Mg_3_Ga_2_SnO_8_ Phosphors for Multifunctional Applications

**DOI:** 10.3390/molecules30091945

**Published:** 2025-04-27

**Authors:** Zaifa Yang

**Affiliations:** College of Physics and Electronic Engineering, Qilu Normal University, Jinan 250200, China; yangzaifa@qlnu.edu.cn; Tel.: +86-1066778147

**Keywords:** optical temperature sensing, energy transfer, phosphor

## Abstract

In recent years, rare earth ion and transition metal ion co-doped fluorescent materials have attracted a lot of attention in the fields of WLEDs and optical temperature sensing. In this study, I successfully prepared the dual-emission Mg_3_Ga_2_SnO_8_:Eu^3+^,Mn^4+^ red phosphors and the XRD patterns and refinement results show that the prepared phosphors belong to the Fd-3m space group. The energy transfer process between Eu^3+^ and Mn^4+^ was systematically investigated by emission spectra and decay curves of Mg_3_Ga_2_SnO_8_:0.12Eu^3+^,yMn^4+^ (0.002 ≤ y ≤ 0.012) phosphors and the maximum value of transfer efficiency can reach 71.2%. Due to the weak thermal quenching effect of Eu^3+^, its emission provides a stable reference for the rapid thermal quenching of the Mn^4+^ emission peak, thereby achieving good temperature measurement performance. The relative thermometric sensitivities of the fluorescence intensity ratio and fluorescence lifetime methods reached a maximum value of 2.53% K^−1^ at 448 K and a maximum value of 3.38% K^−1^ at 473 K. In addition, the prepared WLEDs utilizing Mg_3_Ga_2_SnO_8_:0.12Eu^3+^ phosphor have a high color rendering index of 82.5 and correlated color temperature of 6170 K. The electroluminescence spectrum of the synthesized red LED device by Mg_3_Ga_2_SnO_8_:0.009Mn^4+^ phosphor highly overlaps with the absorption range of the phytochrome P_FR_ and thus can effectively promote plant growth. Therefore, the Mg_3_Ga_2_SnO_8_:Eu^3+^,Mn^4+^ phosphors have good application prospects in WLEDs, temperature sensing, and plant growth illumination.

## 1. Introduction

Phosphor-converted white LEDs (WLEDs) are widely used in solid-state lighting, liquid crystal displays, and the medical industry because of their high luminous efficiency, long lifetime, and environmental friendliness [1,2]. Currently, the main use of gallium nitride (GaN) or indium gallium nitride (GaInN) is in blue semiconductor chips combined with yellow Y_3_Al_5_O_12_:Ce^3+^ phosphor to produce white light [3]. However, it is difficult to meet the conditions for high-quality indoor lighting and backlight display due to insufficient red light [4]. Therefore, the development of stable and efficient red phosphors has important research value. In addition, fluorescence thermometry is a method of measuring temperature by utilizing luminescent properties that are responsive to temperature changes. Fluorescence temperature sensors have a series of advantages over other traditional electrical temperature measurement techniques, such as fast response time, high accuracy, and high spatial and temporal resolution [5,6]. In particular, fluorescence intensity ratio (FIR)- and fluorescence lifetime (FL)-based temperature measurement methods have excellent stability and are less susceptible to emission losses and excitation power fluctuations, thus enabling high-precision and high-resolution temperature measurements [7]. Although fluorescent lighting materials for LEDs and fluorescent temperature sensing materials have been heavily investigated, realizing multifunctional applications with the same material is still a great challenge.

Trivalent rare earth Eu^3+^ ions can be effectively excited by near-ultraviolet (NUV) and blue light due to their special 4f^6^ shell-layer structure, thus displaying strong red emission, which is caused by the ^5^D_0_→^7^F_J_ transitions [8]. Therefore, Eu^3+^ ions are frequently doped into certain matrix materials as activators to emit vivid and enjoyable red light. In contrast, Mn^4+^ ions with a 3d^3^ electronic configuration can exhibit broader excitation and emission bands due to its sensitivity to the coordination environment [9]. The completely different electronic configurations of Eu^3+^ and Mn^4+^ result in different temperature dependence of their luminescence intensity [10]. Therefore, Eu^3+^ and Mn^4+^ co-doped phosphors are ideal materials for designing a novel optical temperature measurement. In recent years, there have been many reports on Eu^3+^ and Mn^4+^ co-doped optical thermometry materials, such as La_2_LiSbO_6_:Eu^3+^,Mn^4+^ [11], Sr_2_InTaO_6_:Eu^3+^,Mn^4+^ [12], Ca_2_GdSbO_6_:Mn^4+^,Eu^3+^ [13], and so on. In addition, the matrix materials are equally important for the photoluminescence and optical thermometry properties of phosphors. Stannate materials are excellent substrates for ions doping due to their excellent stability and unique crystal environment [14]. Mg_3_Ga_2_SnO_8_ (MGS) matrix materials with a cubic structure were first reported by Zhu et al. They constructed MGS substrates based on Mg_2_TiO_4_ through a co-substitution strategy of [Ga^3+^-Ga^3+^] instead of [Mg^2+^-Ti^4+^] and Sn^4+^ instead of Ti^4+^ [15]. The results show that the MGS matrix material has stable physicochemical properties and low phonon energy of the optical mode [16]. Therefore, the study of Eu^3+^ and Mn^4+^ co-doped MGS phosphors for WLEDs and optical temperature measurement has attracted our attention.

In this work, dual-emission MGS:Eu^3+^,Mn^4+^ phosphors with tunable red light emission were successfully prepared. The crystal structure, phase purity, and luminescence properties were comprehensively studied. The results of the X-ray diffractometer (XRD) patterns indicate the successful synthesis of the MGS crystal structure. The systematic analysis of the emission spectra and decay curves of MGS:0.12Eu^3+^,yMn^4+^ (0.002 ≤ y ≤ 0.012) illustrates the existence of energy transfer between Eu^3+^ and Mn^4+^. The maximum efficiency of energy transfer can reach 71.4%. The temperature sensing properties of the MGS:0.12Eu^3+^,0.004Mn^4+^ phosphor were explored in detail based on FIR and FL modes, and higher absolute sensitivity (S_a_) and relative sensitivity (S_r_) were obtained. Moreover, we further completed the package testing of the phosphors for application in LEDs. In conclusion, MGS:Eu^3+^,Mn^4+^ phosphors have potential applications in optical temperature measurement and illumination.

## 2. Results and Discussion

### 2.1. Crystal Structure

**Figure 1:**
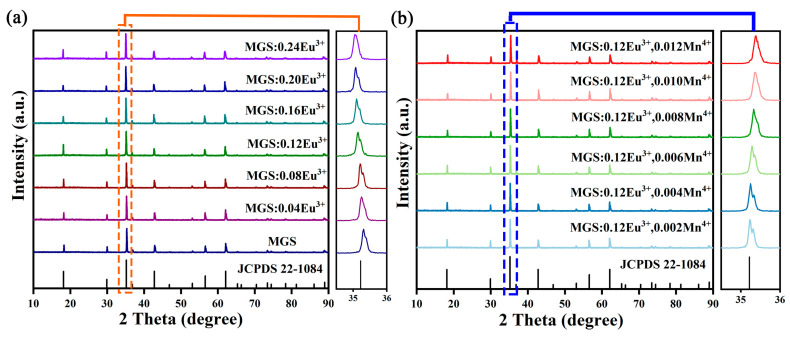
The XRD patterns of (**a**) MGS:xEu^3+^ (0.04 ≤ x ≤ 0.24) and (**b**) MGS:0.12Eu^3+^,yMn^4+^ (0.002 ≤ y ≤ 0.012) samples.

Figure 1a demonstrates the XRD patterns of MGS and MGS:xEu^3+^ (0.04 ≤ x ≤ 0.24) samples. The XRD diffraction peaks of all the samples were sharp and well matched with the MGS standard card (JCPDS 22-1084), indicating that all the synthesized phosphors were well crystallized and in pure phase. Figure 1b also shows the XRD patterns of Mn^4+^ and Eu^3+^ co-doped MGS phosphors and the sharp diffraction peaks of the samples indicate that the prepared phosphor has good crystallinity [17]. In addition, the positions of all the diffraction peaks corresponded to the standard cards of MGS, which further confirmed that the MGS:0.12Eu^3+^,yMn^4+^ (0.002 ≤ y ≤ 0.012) phosphors were pure phase. The main diffraction peak is slightly shifted to the left for the MGS:xEu^3+^ (0.04 ≤ x ≤ 0.24) samples; however, the main diffraction peak is slightly shifted to the right for the MGS:0.12Eu^3+^,yMn^4+^ (0.002 ≤ y ≤ 0.012) samples. Such a result comes mainly from the change in the lattice during ion substitution according to the Bragg equation [18]. Considering the radius similarity and charge balance principle, Eu^3+^ is most likely to take the place of Ga^3+^ and Mn^4+^ is most likely to take the place of Sn^4+^. The corresponding ionic radii of MGS are as follows: Ga^3+^ (r = 0.86 Å, CN = 6); Sn^4+^ (r = 0.62 Å, CN = 6); Eu^3+^ (r = 0.947 Å, CN = 6); and Mn^4+^ (r = 0.53 Å, CN = 6) [19]. Obviously, the larger radius Eu^3+^ occupying the Ga^3+^ site causes the expansion of the cell, while the smaller radius Mn^4+^ occupying the Sn^4+^ site causes the contraction of the cell, and thus the angles are shifted in two different directions in the XRD patterns, respectively. In order to further accurately characterize the lattice positions occupied by the dopant ions, the structure of the samples was further refined.

**Figure 2:**
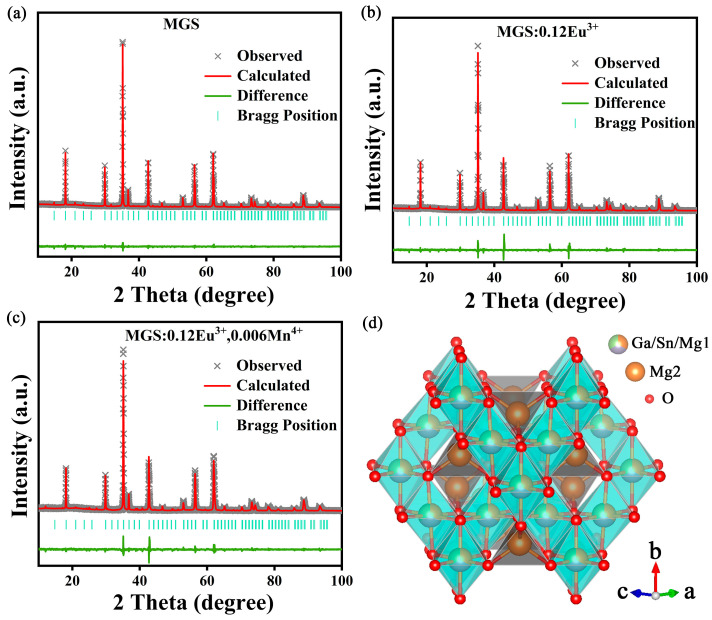
XRD Rietveld refinement of (**a**) MGS (**b**) MGS:0.12Eu^3+^ and (**c**) MGS:0.12Eu^3+^,0.006Mn^4+^. (**d**) Crystal structure of MGS host lattice.

To further investigate the crystal structure data of MGS, MGS:0.12Eu^3+^ and MGS:0.12Eu^3+^,0.006Mn^4+^, the measured XRD result was refined by the EXPGUI version program software of the General Structure Analysis System (GSAS), and the results are shown in Figure 2a–c and Table 1. All refinement fitting parameters—R_p_, R_wp_, and χ^2^—converged to low levels, indicating reliable refinement results [20]. Moreover, the cell volume gradually increases from 604.85 Å^3^ to 605.17 Å^3^ with the addition of Eu^3+^ ions and decreases from 604.85 Å^3^ to 603.27 Å^3^ with the addition of Mn^4+^ ions, which further confirms the successful doping of Eu^3+^ and Mn^4+^. Figure 2d displays the crystal structure of MGS. There are two kinds of Mg^2+^ in the crystal structure, one part of Mg(1)^2+^ combines with eight O^2−^ ions in the form of body-centered cube to form [MgO_6_] octahedra, and the rest of Mg(2)^2+^ combines with four O^2−^ ions to form [Mg(2)O_4_] trihedra. The Mg(1), Ga, and Sn occupy the same position in the lattice and form an octahedral structure with six oxygen atoms. The [Mg(1),Sn,GaO_6_] and [Mg(2)O_4_] are alternately connected by sharing oxygen atoms and thus form the basic skeleton of the cubic structure.

**Figure 3:**
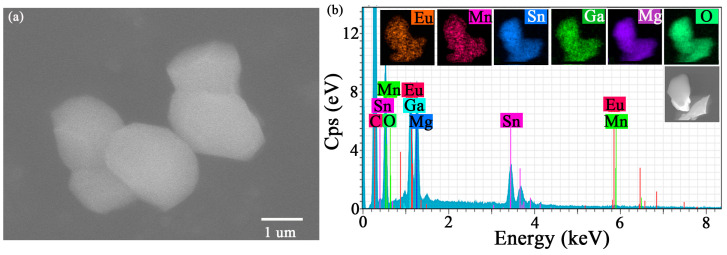
(**a**) The SEM image of MGS:0.12Eu^3+^,0.004Mn^4+^. (**b**) The corresponding element mapping of Eu, Mn, Sn, Ga, Mg, and O in the MGS:0.12Eu^3+^,0.004Mn^4+^ phosphor.

The SEM image of MGS:0.12Eu^3+^,0.004Mn^4+^ is shown in Figure 3a. As can be seen from the figure, the particles of the MGS:0.12Eu^3+^,0.004Mn^4+^ phosphor exhibits irregular shapes without obvious agglomerations, and the particle size distribution ranges from 1 to 2 µm. Figure 3b shows all of the coherent elements in the energy dispersive X-ray (EDX) spectra, and the elemental mass ratios are consistent with the chemical formulas, further demonstrating the successful preparation of the MGS:0.12Eu^3+^,0.004Mn^4+^ sample. The elemental mapping of the representative particles is also shown in Figure 3b. The elemental mapping profiles of the representative particles showed that the elements Eu, Mn, Sn, Ga, Mg, and O were uniformly distributed on the particles without significant elemental aggregation, further indicating that Eu^3+^ ions and Mn^4+^ ions were successfully doped into the MGS matrix.

### 2.2. Optical Properties

**Figure 4:**
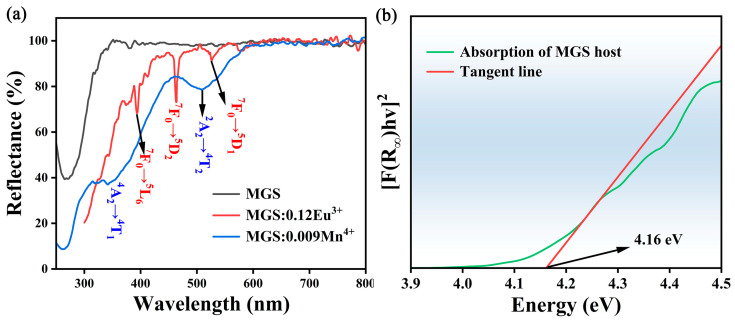
(**a**) The diffuse reflectance spectra of MGS matrix, MGS:0.12Eu^3+^, and MGS:0.009Mn^4+^. (**b**) Energy band structure diagram of MGS.

Figure 4a shows the diffuse reflectance spectra in the UV-visible range for the MGS matrix and the MGS:0.12Eu^3+^ and MGS:0.009Mn^4+^ samples. A highly reflective region appears in the diffuse reflectance curve of the MGS matrix material (350–800 nm) as well as a strong absorption band (200–350 nm). The MGS:0.009Mn^4+^ sample shows two distinct strong absorption bands corresponding to ^4^A_2_-^4^T_1_ and ^4^A_2_-^4^T_2_ transitions [21]. However, the MGS:0.12Eu^3+^ sample shows several absorption peaks in the UV region, and they all come from the f-f transitions of Eu^3+^ [22]. In addition, the band gap (*E_g_*) can be calculated according to Kubelka–Munk formula [23]:

(1) $${\left[h\nu F(R_{\infty })\right]}^{n}=A(h\nu -E_{g})$$

(2) $$F(R)={\left(1-R\right)}^{2}/2R$$

where *F(R)* represents the absorption coefficient, *R* denotes the measured diffuse reflection coefficient, *hν* denotes photon energy, *n* = 2 and 1/2 correspond to the direct and indirect bandgaps, respectively, and A is a constant. According to previous reports, MGS has a direct bandgap, so *n* = 2 [24]. As shown in Figure 4b, the optical bandgap of MGS matrix is estimated to be 4.16 eV based on a linear extrapolation of the function [*F(R_∞_*)*hv*]^2^ = 0. The above results indicate that the MGS matrix has a sufficiently large band gap and is a potential matrix material for the preparation of Eu^3+^ and Mn^4+^ co-doped samples.

**Figure 5:**
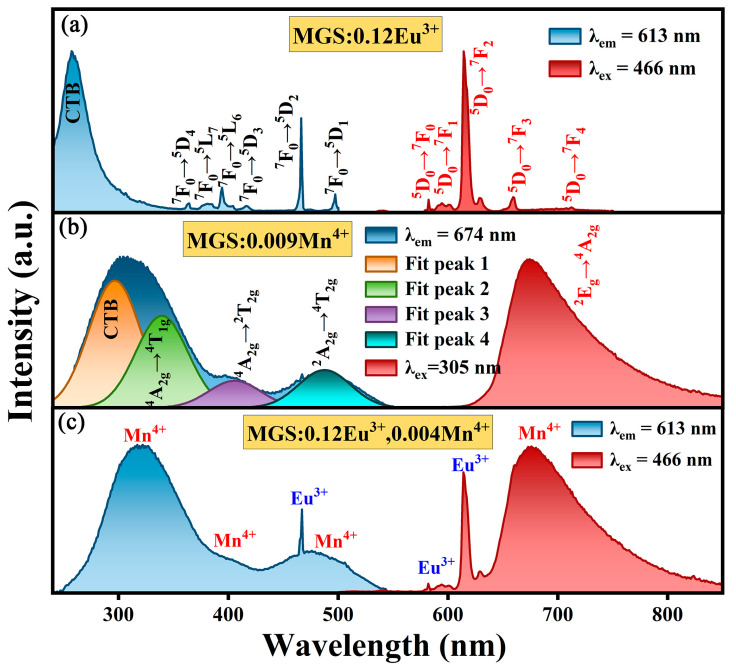
The excitation and emission spectra of (**a**) MGS:0.12Eu^3+^, (**b**) MGS:0.009Mn^4+^, and (**c**) MGS:0.12Eu^3+^,0.004Mn^4+^.

The excitation and emission spectra of MGS:0.12Eu^3+^ are shown in Figure 5a. The strong absorption of the sample between 200 and 320 nm is from the charge transfer band (CTB) between O^2−^→Eu^3+^ [25]. At 362 nm, 381 nm, 394 nm, 416 nm, 466 nm, and 497 nm excitation centers, the Eu^3+^ ions are sharply excited, and the excitation centers are attributed to the f-f characteristic transitions of the Eu^3+^ ions (^7^F_0_→^5^D_4_, ^5^L_7_, ^5^L_6_, ^5^D_3_, ^5^D_2_ and ^5^D_1_) [26]. The ^7^F_0_→^5^D_2_ transition at 466 nm has the highest intensity, indicating that the MGS:0.12Eu^3+^ sample can be effectively excited by blue light. The characteristic peaks at 582, 594, 613, 659, and 710 nm in the emission spectrum belong to the ^5^D_0_→^7^F_J_ (J = 0, 1, 2, 3 and 4) transitions of Eu^3+^ [27]. Figure 5b shows the excitation and emission spectra of MGS:0.009Mn^4+^. The excitation spectrum contains four sets of excitation peaks at 295, 339, 405, and 478 nm after Gaussian split peak fitting. The excitation peak at 295 nm is generated by the CTB from O^2-^→Mn^4+^, while the excitation peaks at 339, 405, and 478 nm are generated by the ^4^A_2_→^4^T_1_, ^2^A_2_→^2^T_2_, and ^2^A_2_→^4^T_2_ transitions of Mn^4+^, respectively [28]. The emission spectrum of MGS:0.009Mn^4+^ exhibits broadband emission centered at 674 nm from the ^2^E→^4^A_2_ transition [29]. Comparing the excitation spectrum of MGS:0.009Mn^4+^ sample with the emission range of MGS:0.12Eu^3+^ phosphor, the overlap between 500 nm and 550 nm suggests the possibility of energy transfer occurring between the Eu^3+^ ions and the Mn^4+^ ions. The excitation and emission spectra of MGS:0.12Eu^3+^,0.004Mn^4+^ are shown in Figure 5c. The emission spectra are mainly at 613 and 674 nm, where the 613 nm band is characterized by the emission of Eu^3+^ ions, while the emission at 674 nm is characterized by the emission of Mn^4+^ ions. Moreover, the characteristic excitation peaks of Eu^3+^ and Mn^4+^ are detected in the same emission peaks. The above results suggest that the energy transfer may occur between Eu^3+^ and Mn^4+^.

**Figure 6:**
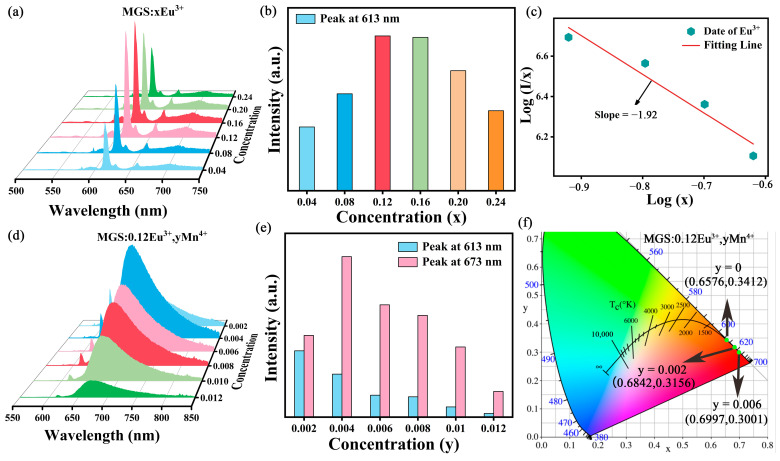
(**a**) The emission spectra of MGS:xEu^3+^ (0.04 ≤ x ≤ 0.24). (**b**) The integrated emission intensity at 613 nm with different concentrations of Eu^3+^. (**c**) The relationship between lg(x) and lg(I/x). (**d**) The emission spectra of MGS:0.12Eu^3+^,yMn^4+^ (0.002 ≤ y ≤ 0.012). (**e**) The integrated emission intensity at 613 nm and 673 nm with concentrations of different Mn^4+^. (**f**) CIE chromaticity coordinates of MGS:0.12Eu^3+^,yMn^4+^ (y = 0, 0.002 and 0.006).

The emission spectra of MGS:xEu^3+^ (0.04 ≤ x ≤ 0.24) phosphors with different Eu^3+^ doping concentrations are given in Figure 6a. There is no significant change in the shape and position of the emission peaks with increasing Eu^3+^ doping concentration. Figure 6b shows the variation in luminescence intensity, and the luminescence intensity reaches the highest when the doping concentration of Eu^3+^ is increased to 0.12, and after that, the luminescence intensity of Eu^3+^ gradually decreases due to the concentration quenching. Based on Dexter’s theory, the mechanism of multipolar interactions was determined by the following equation [30]:

(3) $$\frac{I}{x}=K{\left(1+\beta {\left(x\right)}^{Q/3}\right)}^{-1}$$

where *K* and *β* are substrate-related constants. *Q* = 6, 8, or 10 correspond to dipole–dipole (d-d), dipole–quadrupole (d-q), and quadrupole–quadrupole (q-q) interactions [31]. The lg(*I/x*)~lg(*x*) relationship for MGS:xEu^3+^ phosphors is illustrated in Figure 6c, and the linear fit yields a slope of −1.92, so *Q* = 5.76, implying that d-d interaction is the main mechanism leading to concentration quenching.

Figure 6d shows the emission spectra of MGS:0.12Eu^3+^,yMn^4+^ (0.002 ≤ y ≤ 0.012) samples. Figure 6e shows the variation in normalized emission intensity of MGS:0.12Eu^3+^,yMn^4+^ (0.002 ≤ y ≤ 0.012). As the concentration of Mn^4+^ increases, the emission intensity of Eu^3+^ at 613 nm shows a monotonically decreasing trend, whereas the emission intensity of Mn^4+^ at 673 nm first gradually increases and then decreases. This result comes from the enhancement of energy transfer between Eu^3+^ and Mn^4+^ with the increase of Mn^4+^ doping concentration [32] and the decrease in strength of Mn^4+^ mainly comes from the concentration quenching of Mn^4+^. To further investigate the multicolor tunable emission in MGS:0.12Eu^3+^,yMn^4+^ (0.002 ≤ y ≤ 0.012) phosphors, the CIE coordinates of MGS:0.12Eu^3+^,yMn^4+^ (0.002 ≤ y ≤ 0.012) phosphors were plotted in Figure 6f, from which a color change in the CIE coordinates from red to deep red can be clearly observed. At the same time, the color coordinates of all the samples are at the edge of the coordinate chart, indicating that the samples have very high color purity of light emission, which provides the conditions for the later LED packaging application.

**Figure 7:**
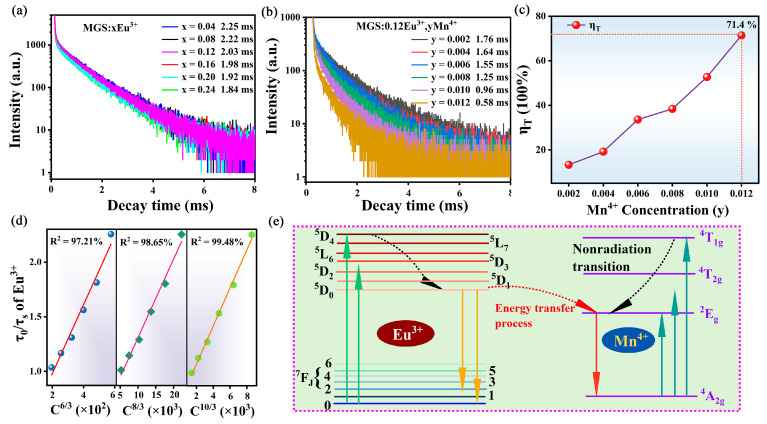
The decay curves of (**a**) MGS:xEu^3+^ (0.04 ≤ x ≤ 0.24) and (**b**) MGS:0.12Eu^3+^,yMn^4+^ (0.002 ≤ y ≤ 0.012) phosphors. (**c**) The energy transfer efficiency of MGS:0.12Eu^3+^,yMn^4+^ (0.002 ≤ y ≤ 0.012). (**d**) Dependence of lifetime ratio τ_S0_/τ_S_ of Eu^3+^ on C^6/3^, C^8/3^, and C1^0/3^. (**e**) Energy level diagrams of Eu^3+^ and Mn^4+^.

Figure 7a shows the decay curves of MGS:xEu^3+^ (0.04 ≤ x ≤ 0.24) samples. The value of fluorescence lifetime can be obtained by fitting a double exponential equation as follows [33]:

(4) $$I=I_{0}+A_{1}\exp(-t/\tau_{1})+A_{2}\exp(-t/\tau_{2})$$

The average lifetime of a sample can be calculated using the following formula [34]:

(5) $$\tau =({A_{1}\tau_{1}}^{2}+{A_{2}\tau_{2}}^{2})/(A_{1}\tau_{1}+A_{2}\tau_{2})$$

where *τ_1_* and *τ_2_* represent the fast and slow parts of the lifetime, *I_t_* denotes the integrated emission intensity at time t, and *A_1_* and *A_2_* are constants, respectively. The average lifetime of MGS:xEu^3+^ (0.04 ≤ x ≤ 0.24) decreases monotonically from 2.25 ms to 1.84 ms as the Eu^3+^ doping increases from 0.04 to 0.24. The main reasons for the lifetime attenuation are that the spacing between dopant ions decreases with increasing concentration, leading to the enhanced probability of non-radiative transitions [35]. To further demonstrate the occurrence of energy transfer, the fluorescence decay curves of Eu^3+^ ions in MGS:0.12Eu^3+^,yMn^4+^ (0.002 ≤ y ≤ 0.012) are shown in Figure 7b. The average lifetimes *τ* of MGS:0.12Eu^3+^,yMn^4+^ (0.002 ≤ y ≤ 0.012) were calculated from Equation (6) as 1.76, 1.64, 1.55, 1.25, 0.96, and 0.58 ms. Such results provide further evidence that energy transfer from Eu^3+^ ions to Mn^4+^ ions occurs in MGS:0.12Eu^3+^,yMn^4+^ (0.002 ≤ y ≤ 0.012) samples. The following equation can be used to analyze the energy transfer efficiency [36]:

(6) $$\eta_{T}=1-\tau_{S}/\tau_{S0}$$

where *τ_S_* and *τ_S0_* are the fluorescence lifetimes of Eu^3+^ at single doping and co-doping, respectively. Figure 7c demonstrates the relationship between the Mn^4+^ ion concentration and the energy transfer efficiency. The energy transfer efficiency reaches 71.4% at the Mn^4+^ ion doping concentration of 0.012. The multipole–multipole interactions can be used to analyze energy transfer mechanism according to Dexter’s theory [37]:

(7) $$\tau_{S0}/\tau_{S}\propto C^{n/3}$$

where *C* is the total doping concentration of Eu^3+^ and Mn^4+^ ions and n is 6, 8, and 10 corresponding to the three types of interactions: d-d, d-q, and q-q interactions, respectively. The best linear fit value of n is 10 and the correlation fitting exponent R^2^ is 0.9948, as shown in Figure 7d. This result suggests that the energy transfer mechanism between Eu^3+^ ions and Mn^4+^ ions in the MGS:0.12Eu^3+^,yMn^4+^ (0.002 ≤ y ≤ 0.012) samples is mainly a q-q interaction. Figure 7e displays the schematic diagram of Eu^3+^ to Mn^4+^ in the MGS matrix. When MGS:0.12Eu^3+^,yMn^4+^ (0.002 ≤ y ≤ 0.012) samples were excited by blue light, the ground state electrons of Eu^3+^ ions were lifted to higher excited states. After that, the excited state electrons of Eu^3+^ rapidly relax radiationally to a lower excited state. Then, some of the electrons radiatively return from ^5^D_0_ to ^7^F_J_ (J = 0, 1, 2, 3, 4 and 5), releasing the characteristic emission of Eu^3+^. The other excited state electrons come to the excited state of Mn^4+^ through an energy transfer process, and then leap back to the ^4^A_2_ state, releasing the characteristic emission of Mn^4+^.

**Figure 8:**
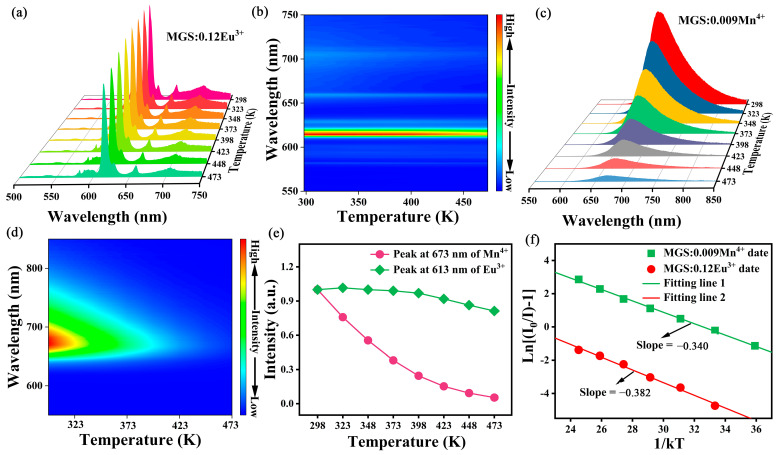
(**a**) Temperature-dependent emission spectra and (**b**) two-dimensional fluorescence topographical mapping of MGS:0.12Eu^3+^. (**c**) Temperature-dependent emission spectra and (**d**) two-dimensional fluorescence topographical mapping of MGS:0.009Mn^4+^. (**e**) The integrated intensities of Mn^4+^ at 673 nm and Eu^3+^ at 613 nm. (**f**) The plot of ln[(I_0_/I_(T)_-1] VS 1/KT for MGS:0.009Mn^4+^ and MGS:0.12Eu^3+^ phosphors.

Thermal stability is an important parameter for evaluating luminescent performance and commercial availability. The temperature-dependent emission spectra and relative emission contour spectra of the MGS:0.12Eu^3+^ phosphor are displayed in Figure 8a,b and the temperature-dependent emission spectra and relative emission contour spectra of the MGS:0.009Mn^4+^ phosphor at different temperatures are displayed in Figure 8c,d. The peak shape and peak position of the MGS:0.12Eu^3+^ and MGS:0.009Mn^4+^ phosphors remained essentially unchanged with increasing temperature. Luminous intensity will be reduced accordingly and the temperature increase will intensify the phosphor luminescence center electron non-radiative transition. Figure 8e displays the normalized intensity plots of the characteristic emission peaks of Eu^3+^ (613 nm) in the MGS:0.12Eu^3+^ sample and Mn^4+^ (673 nm) in the MGS:0.009Mn^4+^ as the function of temperature. The emission intensity of the MGS:0.12Eu^3+^ phosphor at 423 K can still maintain 91.84% of the highest emission intensity at room temperature, which is superior to the majority of the reported Eu^3+^-activated red phosphors, such as: Sr_2_LaNbO_6_:Eu^3+^ (62.99%@423 K) [38], Ba_2_GdSbO_6_:0.5Eu^3+^ (79.27%@423 K) [39], and Li_6_SrLa_2_Nb_2_O_12_:Eu^3+^ (82.25%@423 K) [40]. The result suggests that the synthesized MGS:0.12Eu^3+^ is a promising red phosphor that is expected to be applied in WLEDs for lighting. In comparison, MGS:0.009Mn^4+^ phosphor exhibits poor thermal stability. In addition, the activation energy (*E_a_*) can be calculated using the Arrhenius equation [41]:

(8) $$I_{\left(T\right)}=\frac{I_{0}}{1+cexp(-(E_{a}/kT))}$$

where *I_0_* denotes the luminous intensity of the phosphor at room temperature, *I_(T)_* denotes the luminous intensity of the phosphor at different temperatures, *c* is a constant, *k* is Boltzmann’s constant, and *T* is the temperature of the test environment. Figure 8f shows the linear fit of ln[(I_0_/I)-1] versus 1/kT for the MGS:0.12Eu^3+^ and MGS:0.009Mn^4+^ samples. According to the slopes of linear fit, the value of *E_a_* for MGS:0.009Mn^4+^ is 0.340 eV, while the value of *E_a_* for MGS:0.12Eu^3+^ is 0.382 eV.

**Figure 9:**
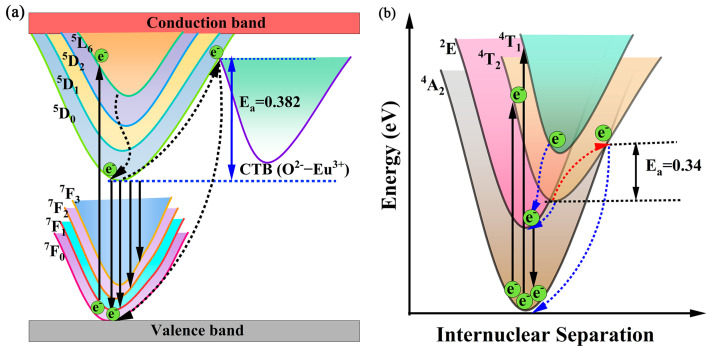
The configurational coordinate diagram for the (**a**) Eu^3+^ ions and (**b**) Mn^4+^ ions.

To further explain the process of thermal quenching, bit pattern coordinate diagrams can be used. As shown in Figure 9a, the electrons in the ^7^F_J_ state are excited to the ^5^D_J_, ^5^L_6_, or CTB states excited by UV or blue light. After non-radiative relaxation of electrons from the upper excited state to the ^5^D_0_ state, most of the electrons will radiatively jump from the ^5^D_0_ state to the ground state ^7^F_J_ and then emit red light. At room temperature, most electrons in the CTB state can overcome the energy potential barrier to reach the ^5^D_0_ state under electron–phonon coupling and then radiatively jump to the ground state ^7^F_J_. As the temperature increases, electrons in the excited state may overcome the low-energy potential barrier with phonon assistance, reach the intersection with the ground state from the excited state, and then non-radiatively relax to the ground state equilibrium position, resulting in thermal quenching [42]. As shown in Figure 9b, when Mn^4+^ is excited by UV light, electrons are pushed from the ground state (^4^A_2_) to the first excited level (^2^E) and higher excited states (^4^T_2_ or ^4^T_1_). At room temperature, electrons in higher excited states drop to the first excited state (^2^E) via nonradiative relaxation, and subsequently to the ground state (^4^A_2_) with radiative relaxation with a deep red emission. However, with the influence of phonon interaction at high temperatures, electrons in the first excited state (^2^E) can be thermally generated and released through the crossing point between ^4^T_2_ and ^4^A_2_ by nonradiative relaxation in the configuration coordinate diagram, leading to the formation of the thermal quenching phenomenon.

### 2.3. Optical Temperature Sensing

**Figure 10:**
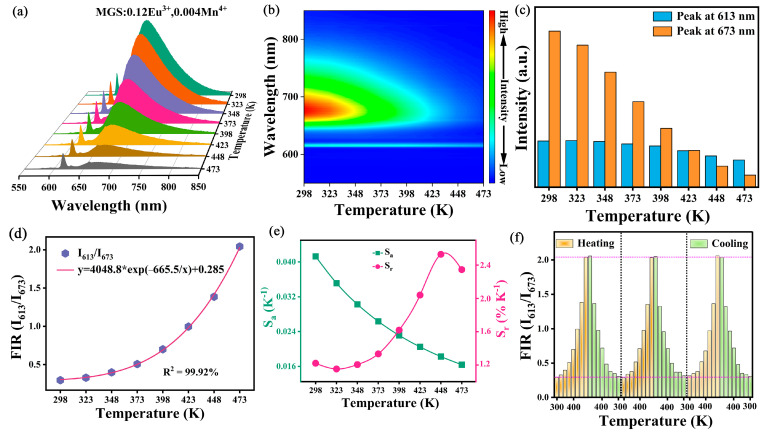
(**a**) The temperature-dependent emission spectra and (**b**) the corresponding contour maps of MGS:0.12Eu^3+^,0.004Mn^4+^. (**c**) The integrated intensities of MGS:0.12Eu^3+^,0.004Mn^4+^ at 613 nm and 673 nm. (**d**) Temperature-dependent FIR values from I_613_/I_673_ of MGS:0.12Eu^3+^,0.004Mn^4+^. (**e**) Calculated S_r_ and S_a_ at different temperatures by FIR. (**f**) FIR temperature-cycling values of I_613_/I_673_ with three cycles.

To further explore this phosphor in optical temperature sensing applications, the quantitative relationship between the fluorescence intensity of different emission peaks and the temperature interdependence can be utilized for exploratory studies. Figure 10a,b show the temperature-dependent emission spectra and relative emission contour spectra of the MGS:0.12Eu^3+^,0.004Mn^4+^ phosphor at temperatures ranging from 298 K to 473 K. Figure 10c shows the normalized intensity plots of the characteristic emission peaks of Eu^3+^ (613 nm) and Mn^4+^ (673 nm) in the MGS:0.12Eu^3+^,0.004Mn^4+^ sample at different temperatures. The luminescence at 673 nm of Mn^4+^ bursts faster, while the emission intensity at 613 nm of Eu^3+^ bursts slower with the increase in temperature. Since the intensities of the Eu^3+^ and Mn^4+^ emission peaks respond differently to temperature changes, they can be used to design non-contact optical thermometers based on the FIR technique. The FIR can be calculated according to the following equation [43]:

(9) $$FIR=\frac{I_{613nm}}{I_{673nm}}\approx A\exp(-B/T)+C$$

where *A*, *B*, and *C* are constants and ΔE is the energy gap. Figure 10d shows the relationship between the *FIR* (*I*_613 nm_/*I*_673 nm_) value and temperature, which can be expressed by the fitting result as FIR = 4048.8 × exp(−665.5/T) + 0.285. The *S_a_* and *S_r_* can be determined according to the following equations [44]:

(10) $$S_{a}=\left|\frac{\partial FIR}{\partial T}\right|$$

(11) $$S_{r}=100\%\left|\frac{\partial FIR}{FIR\partial T}\right|$$

Figure 10e shows the fitted curves of *S_a_* and *S_r_* as a function of temperature calculated by the above equations. The value of S_a_ shows a decreasing trend with increasing temperature and has a maximum value of 0.041 K^−1^ at 298 K. The value of *S_r_* shows an increasing and then a decreasing trend and reaches a maximum value of 2.53% K^−1^ at 448 K. Compared to the optical thermometry phosphors already reported in Table 2, the MGS:0.12Eu^3+^,0.004Mn^4+^ phosphor has a wider thermometry range and higher sensitivity. To better analyze the cyclic stability, the change in FIR value with temperature during multiple temperature cycling is shown in Figure 10f, and the FIR value is able to recover to the initial state after three temperature cycles, which indicates that the phosphor has good reversibility and reliability in temperature sensing. In conclusion, the MGS:0.12Eu^3+^,0.004Mn^4+^ phosphor has good temperature sensing performance and can be regarded as an optical material with potential value for further research in the field of optical temperature sensing.

**Figure 11:**
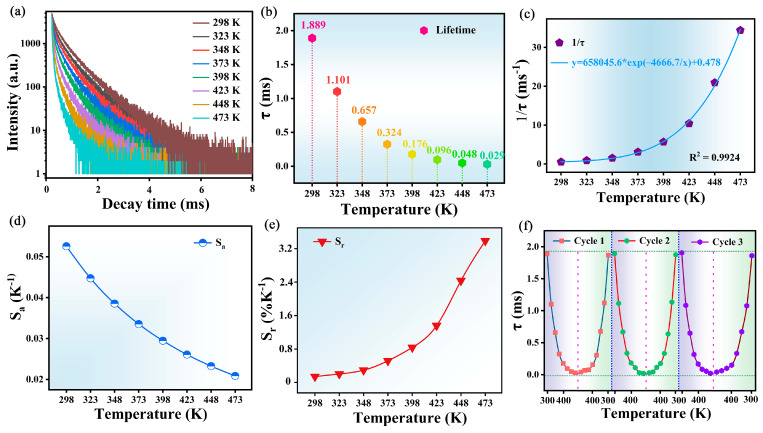
(**a**) The decay curves of MGS:0.12Eu^3+^,0.004Mn^4+^ at the temperatures range from 298 K to 473 K. (**b**) The lifetime of Mn^4+^ for MGS:0.12Eu^3+^,0.004Mn^4+^ sample at different temperatures. (**c**) The fitting curve of temperature-dependent FL. The (**d**) S_a_ and (**e**) S_r_ based on FL of Mn^4+^. (**f**) The lifetime of Mn^4+^ at different temperatures with three cycles.

Temperature sensing using fluorescence lifetimes is another very promising measurement option with the inherent advantage of calibration-free measurements that are not affected by external factors such as sample size and excitation power fluctuations. Figure 11a displays the decay curves for the MGS:0.12Eu^3+^,0.004Mn^4+^ phosphor at different temperatures. The luminescence lifetime of Eu^3+^ decays from 1.889 ms to 0.025 ms with increasing temperature, as shown in Figure 11b, and the variability of the luminescence lifetime can be fitted by an Arrhenius-type equation with the following equation [45]:

(12) $$\frac{1}{\tau_{\left(T\right)}}=\frac{1}{\tau_{0}}(1+D\exp(-\Delta E’/(kT))$$

where *τ_(T)_* and *τ_0_* denote the Eu^3+^ luminescence lifetime at test temperature *T* and room temperature, respectively. As shown in Figure 11c, the relationship between the FL values and temperature can be expressed by the fitting result as 1/τ = 65,8045.6 × exp(−4666.7/T) + 0.478. To assess the feasibility of FL as a model for thermometry parameters, the *S_a_* and *S_r_* can be calculated using the following equations [46]:

(13) $$S_{a}=\left|\frac{\partial \tau }{\partial T}\right|$$

(14) $$S_{r}=100\%\left|\frac{1}{\tau }\frac{\partial \tau }{\partial T}\right|$$

The *S_a_* value of the MGS:0.12Eu^3+^,0.004Mn^4+^ sample decreases with increasing temperature and possesses a maximum value of 0.053 K^−1^ at 298 K, as shown in Figure 11d. The *S_r_* values of the MGS:0.12Eu^3+^,0.004Mn^4+^ sample show an increasing trend, with a maximum S_r_ of 3.38% K^−1^ at 473 K, as shown in Figure 11e. The MGS:0.12Eu^3+^,0.004Mn^4+^ sample has superior temperature sensitivity properties compared to the temperature parameters of the recently reported co-doped fluorescent materials in Table 2. For optical temperature sensing materials, the reproducibility of FL with temperature is also a prerequisite for the material to be practically applicable. In order to verify the reproducibility of the sample, Figure 11f demonstrates the FL of the MGS:0.12Eu^3+^,0.004Mn^4+^ phosphor, with the temperature firstly increasing and then decreasing for three cycles. It can be observed that the phosphor exhibits good reproducibility of temperature measurement and has potential for practical application.

**Table 2:** S_r_-Max of some co-doped phosphors based on FIR or FL mode.

Sample	Temperature Range (K)	S_r_-Max (% K^−1^)	Mode	Ref.
Ca_2_YZr_2_Al_3_O_12_:Bi^3+^,Eu^3+^	297–573	0.664	FIR	[22]
Sr_3_TaGa_3_Si_2_O_14_:Tb^3+^,Eu^3+^	298–498	0.760	FIR	[26]
La_2_LiSbO_6_:Eu^3+^,Mn^4+^	303–523	0.89	FIR	[11]
Ca_2_LaNbO_6_:Eu^3+^,Mn^4+^	298–498	1.51	FIR	[47]
BaLaMgNbO_6_:Dy^3+^,Mn^4+^	230–470	1.82	FIR	[48]
MGS:0.12Sm^3+^,0.004Mn^4+^	298–473	2.53	FIR	This work
SrGdLiTeO_6_:Sm^3+^, Mn^4+^	298–573	1.30	FL	[8]
Ca_2_GdSbO_6_:Mn^4+^,Eu^3+^	303–523	1.47	FL	[13]
Ba_2_GdNbO_6_:Eu^3+^, Mn^4+^	303–483	1.73	FL	[49]
La_2_MgTiO_6_:Dy^3+^,Mn^4+^	303–503	2.31	FL	[50]
MGS:0.12Sm^3+^,0.004Mn^4+^	298–473	3.38	FL	This work

### 2.4. Applications in LEDs

**Figure 12:**
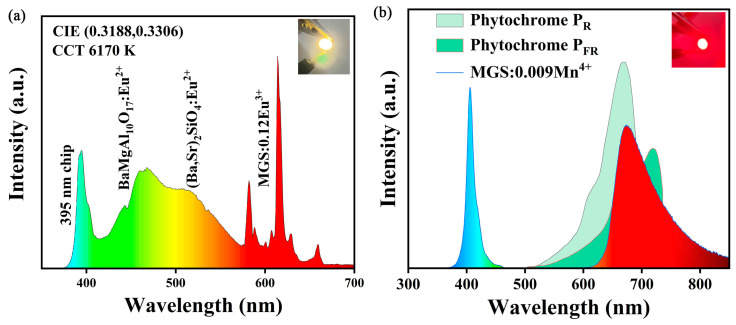
(**a**) The electroluminescence spectrum of the prepared WLED. (**b**) The electroluminescence spectra of the MGS:0.009Mn^4+^ sample encapsulated with a 410 nm UV chip compared to the absorption spectra of P_R_ and P_FR_.

The MGS:0.12Eu^3+^ sample and commercial phosphors BaMgAl_10_O_17_:Eu^2+^ and (Ba,Sr)_2_SiO_4_:Eu^2+^ were encapsulated in the 395 nm commercial chip to make a WLED light source, and its electroluminescence spectrum is shown in Figure 12a. The inset is a photo of this WLED after it is illuminated. The correlated color temperature of the prepared WLED device is 6170 K, and the color rendering index reaches 82.5, which indicates that the MGS:0.12Eu^3+^ phosphor has great potential for use in pc-LED backlit displays. The phytochrome has a specific absorption spectrum, so the wavelength at which the emission spectrum of the phosphor is located needs to be within the absorption spectrum of the phytochrome in order to promote plant photosynthesis more effectively [51]. Figure 12b displays the electroluminescence spectrum of the MGS:0.009Mn^4+^ sample and absorption spectra of the plant photosensitive pigments P_R_ and P_FR_. Obviously, the strongest peak located at 673 nm is highly overlapped with spectrum of P_FR_ and is far away from the peak wavelength of the absorption spectrum of the P_R_. This suggests that the sample MGS:0.009Mn^4+^ can promote the process of energy absorption by the plant photosensitive pigment P_FR_, which in turn selectively promotes some specific processes of plant growth.

## 3. Materials and Methods

### 3.1. Preparation of Materials

A series of MGS:xEu^3+^ (0.04 ≤ x ≤ 0.24) and MGS:0.12Eu^3+^,yMn^4+^ (0.002 ≤ y ≤ 0.012) samples were prepared by adopting a high-temperature solid phase method. Firstly, I weighed the raw materials—(MgCO_3_)_4_·Mg(OH)_2_·5H_2_O (99.5%, Aladdin), Ga_2_O_3_ (99.99%, Aladdin), SnO_2_ (99.5%, Aladdin), Eu_2_O_3_ (99.99%, Aladdin) and MnCO_3_ (99.5%, Aladdin)—according to the designed stoichiometric ratios, and ground and stirred them well in an agate mortar. After thorough grinding and homogenization, the mixed powders were transferred to a crucible of alumina, followed by sintering in the muffle furnace at 1450 °C for 6 h, with air as the sintering atmosphere. Finally, the samples were crushed into powder and subjected to subsequent performance testing.

### 3.2. Characterization of Materials

The XRD data were measured by a Bruker-D8 ADVANCE diffractometer (BRUKER, Germany). The morphology and dimensions of the samples were observed using a field emission scanning electron microscope (SEM, HITACHI, SU8100, Tokyo, Japan). The diffuse reflectance spectra were tested using a spectrophotometer (Pulsar, TU1950, Beijing, China) equipped with an integrating sphere accessory. The excitation and emission spectra of the samples were measured using a FLS1000 fluorescence spectrometer from (Edinburgh, UK). The excitation light source was a xenon lamp, and a variable-temperature accessory was used for variable-temperature testing. For fluorescence lifetime measurement, a microsecond lamp was selected as the light source. The electrochromic performance of packaged light-emitting devices was measured using the Starspec SSP6612 LED photoelectric measuring system (Hangzhou, China).

### 3.3. Preparation of LEDs

The prepared MGS:0.12Eu^3+^ red phosphor was thoroughly and evenly mixed with the commercial blue phosphor BaMgAl_10_O_17_:Eu^2+^ and the commercial green phosphor (Ba, Sr)_2_SiO_4_:Eu^2+^ according to a mass ratio of 10:2:1. Subsequently, this mixture was mixed with the organic silica gel according to a mass ratio of 1:0.7, and then applied onto a NUV chip with a wavelength of 395 nm and encapsulated to form a white light LED device. A single red LED was fabricated by combining the as-prepared MGS:0.009Mn^4+^ phosphor and organic silica gel with a 410 nm InGaN chip.

## 4. Conclusions

In summary, I successfully synthesized MGS:Eu^3+^,Mn^4+^ phosphors with dual emission centers. The XRD, morphology, photoluminescence properties, energy transfer process and temperature-sensitive properties of MGS:Eu^3+^,Mn^4+^ samples have been investigated. The results show that the energy transfer of Eu^3+^→Mn^4+^ ions exists in MGS:Eu^3+^,Mn^4+^ phosphors, and the maximum value of transfer efficiency can reach 71.2%. The CIE chromaticity coordinate plots of the samples show that the effective adjustment of the luminescence color of the double-doped phosphor can be achieved by changing the doping concentration of Mn^4+^. Thanks to the weak thermal burst effect of Eu^3+^, its emission provides a stable reference for the fast thermal quenching of the Mn^4+^ stokes emission peak, which leads to a good thermometry performance. The relative thermometric sensitivities of the two methods reached a maximum value of 2.53% K^−1^ at 448 K and a maximum value of 3.38% K^−1^ at 473 K. The prepared LED lamps have a high color temperature (the relevant color temperature is 6170 K) and a color rendering index R_a_ of 82.5, indicating that the prepared phosphors have good luminescence performance and can be applied to the field of LED lighting.

## Figures and Tables

**Table 1:** The detailed refinement results of MGS, MGS:0.009Mn^4+^, MGS:0.12Eu^3+^, and MGS:0.12Eu^3+^,0.006Mn^4+^ samples.

Sample	MGS	MGS:0.009Mn^4+^	MGS:0.12Eu^3+^	MGS:0.12Eu^3+^,0.006Mn^4+^
Space group	Fd-3m	Fd-3m	Fd-3m	Fd-3m
Symmetry	cubic	cubic	cubic	cubic
a/b/c, Å	8.4570	8.4552	8.4585	8.4530
V, Å^3^	604.85	604.46	605.17	603.99
Z	8	8	8	8
α = β = γ °	90	90	90	90
R_wp_	8.5	9.6	8.8	7.5
R_p_	6.7	7.5	6.6	5.2
χ^2^	2.25	2.83	2.45	2.38

## Data Availability

The more research data are available from the authors on request.
